# Ethics of access to newly approved expensive medical treatments: multi-stakeholder dialogues in a publicly funded healthcare system

**DOI:** 10.3389/fphar.2023.1265029

**Published:** 2024-01-29

**Authors:** Charlotte H. C. Bomhof, Jilles Smids, Sybren Sybesma, Maartje Schermer, Eline M. Bunnik

**Affiliations:** Department of Medical Ethics, Philosophy and History of Medicine, Erasmus MC, Rotterdam, Netherlands

**Keywords:** empirical bioethics, stakeholder engagement, access to expensive treatments, healthcare policy, ethics

## Abstract

**Background:** Due to rising healthcare expenditures, countries with publicly funded healthcare systems face challenges when providing newly approved expensive anti-cancer treatments to all eligible patients. In the Netherlands in 2015, the so-called Coverage Lock (CL), was introduced to help safeguard the sustainability of the healthcare system. Since then, newly approved treatments are no longer automatically reimbursed. Previous work has shown that as policies for access to CL treatments are lacking, patient access to non-reimbursed treatments is limited and variable, which raises ethical issues. The ethics of access were discussed in a series of multi-stakeholder dialogues in the Netherlands.

**Methods:** Three dialogues were held in early 2023 and included physicians, health insurers, hospital executives, policymakers, patients, citizens, and representatives of pharmaceutical companies, patient and professional organizations. In advance, participants had received an ‘argument scheme’ featuring three models: 1) access based on third-party payment (e.g., by pharmaceutical companies, health insurers or hospitals) 2) access based on out-of-pocket payments by patients 3) no access to CL treatments. During the dialogues, participants were asked to discuss the merits of the ethical arguments for and against these models together, and ultimately to weigh them. The discussions were audio-taped, transcribed, coded, and thematically analyzed.

**Results:** Generally, most stakeholders were in favour of allowing access–at least when treatments are clearly beneficial–to treatments in the CL. When discussing third-party payment, stakeholders favoured payment by pharmaceutical companies over payment by health insurers or hospitals, not wanting to usurp collective funds while cost-effectiveness assessments are still pending. Largely, stakeholders were not in favour of out-of-pocket payments, emphasizing solidarity and equal access as important pillars of the Dutch healthcare system. Recurrent themes included the conflict between individual and collective interests, shifting attitudes, withholding access as a means to put pressure on the system, and the importance of transparency about access to CL-treatments.

**Conclusion:** Policies for access to non-reimbursed treatments should address stakeholders’ concerns regarding transparency, equal access and solidarity, and loss of potential health benefits for patients. Multi-stakeholder dialogues are an important tool to help inform policy-making on access to newly approved (too) expensive treatments in countries facing challenges to the sustainability of healthcare systems.

## 1 Introduction

Due to rising healthcare expenditures and a proliferation of expensive medical treatments, countries with publicly funded healthcare systems face challenges when providing newly approved expensive anti-cancer treatments to all eligible patients. As healthcare budgets are limited, increasing use of expensive treatments can lead to the crowding out of other types of healthcare (Rekenkamer, 2020). To safeguard the financial sustainability of healthcare systems, countries apply a range of policies (Stadhouders et al., 2019). As an example of a policy aimed at containing the cost of new expensive treatments, last year, Germany changed the law to reduce the period in which new treatments are reimbursed at the list price from twelve to 6 months. Thus, the price that is negotiated on the basis of health technology assessment will (retroactively) apply after six instead of 12 months, which saves costs (Koyncu, 2022). In the Netherlands in 2015, the so-called Coverage Lock (CL) was introduced to safeguard a sustainable healthcare system (Kleijne, 2016). Since then, newly approved expensive treatments entering the market are no longer automatically reimbursed (see Box 1), which delays patient access to these treatments. Until now, the ethical implications of CL have not been systematically evaluated. In this study, the ethics of access to treatments placed in the CL were discussed in a series of multi-stakeholder dialogues in the Netherlands. As stakeholder engagement is essential for responsible development and implementation of policies (OECD, 2021), more insight into stakeholders’ perspectives regarding access to non-reimbursed treatments is urgently needed, especially in countries with publicly funded healthcare systems.

Box 1 **The healthcare system and Coverage Lock in the Netherlands**

The Netherlands is a country with a publicly funded healthcare system, based on solidarity, granting comprehensive healthcare for all patients (Zorginstituut Nederland, 2022). In practice, this means that all citizens have a mandatory health insurance, which provides them access to all medically necessary care that is reimbursed within the basic healthcare package. Some treatments which are newly approved by the European Medicines Agency (EMA) are not immediately reimbursed within the basic healthcare package, but first placed in the CL (Zorginstituut Nederland, 2020). A treatment is placed in the lock if it has a budget impact exceeding 20 million euros a year for all patients with the disease for which it is prescribed, or if it costs 50,000 euros or more per patient with total costs exceeding a budget impact of 10 million euros a year for one disease. While treatments are in the CL, the Dutch Healthcare Institute issues an advice whether to include the treatment in the basic healthcare package–based on the four criteria efficacy, cost-effectiveness, feasibility and necessity–and when necessary, the Dutch Ministry of Health, Welfare and Sports negotiates with the pharmaceutical company regarding the price. Since the instalment of the CL in 2015, 57 treatment indications have been assessed in the lock (Zorginstituut Nederland, 2023). In July 2023, the Ministry of Health, Welfare and Sports lowered the threshold of the total budget impact of treatments to enter the CL from 40 to 20 million euros (Kuipers, 2023), which means that from July 2023 onwards, an increasing number of newly approved treatments will be placed in the CL. In 2021 and 2022, treatments spent–on average–510 days in the CL (Vereniging Innovatieve Geneesmiddelen, 2023). Most treatments that come out of the CL are included in the basic healthcare package at undisclosed prices. In March 2023, however, for the first time since the introduction of the CL in 2015, negotiations were unsuccessful: Trodelvy, a third-line treatment for triple-negative breast cancer which gives approximately 5.4 months life-prolongation and costs 68,707 euros per patient per treatment, was not included in the basic healthcare package. The Dutch Healthcare Institute recommended inclusion into the basic healthcare package only if the pharmaceutical company would agree to a price reduction of 75%, which the pharmaceutical company did not (Rijksoverheid, 2023b). Also Libmeldy, a treatment for the rare genetic disorder Metachromatic Leukodystrophy, was not included in the basic healthcare package after unsuccessful price negotiations (Rijksoverheid, 2023a). Currently, there are no policies in the Netherlands regarding access to CL treatments, and it is unclear whether patients are able to access treatments that are not (yet) included in the basic healthcare package.

While treatments are in the CL, health insurers have no obligation to reimburse them. Pharmaceutical companies are allowed–but likewise, not obliged–to provide the treatments to patients free of charge through managed access programs. In the period 2015–2020, many pharmaceutical companies did provide managed access to treatments in the CL (Barjesteh van Waalwijk van Doorn-Khosrovani et al., 2021). However, it is unclear for how many patients or in how many hospitals access was possible. A previous interview study amongst a diverse group of Dutch stakeholders regarding access to Nusinersen while it was in the lock, showed that stakeholders perceived the time which treatments spent in the lock to be too long (Scheijmans et al., 2022). Another interview study amongst Dutch physicians showed that physicians sometimes encounter problems when they want to prescribe treatments which are in the CL (Bomhof et al., 2022). This study also showed differences in physicians’ practices: while some physicians tried to arrange access to non-reimbursed treatments for patients, for instance by asking the hospital to fund the treatment, apply for leniency by insurance companies, or look for managed access programs, other physicians did not, because it would take too much time, would involve a lot of administrative work, or because they expected that their application would not be granted. Therefore, it seems that patient access to treatments which are in the CL is sometimes limited and variable in the Netherlands. This raises ethical questions regarding equal access to CL-treatments. As it is expected that the number of CL-treatments will increase in the near future, the need for policies safeguarding fair access to CL-treatments is becoming more urgent.

In this paper, we report on the methods and results of a series of multi-stakeholder dialogues we conducted, which included physicians, health insurers, hospital executives, policymakers, patients, citizens, and representatives of pharmaceutical companies, patient organizations and professional organizations, regarding three policy options or ‘models’ for access to treatments in the CL: 1) access based on third-party payment (e.g., by pharmaceutical companies, health insurers or hospitals) 2) access based on out-of-pocket payments by patients 3) no access to non-reimbursed treatments. These ‘models’ are descriptions of the various possible access routes. Depending on how these access models are (morally) evaluated, the need may arise to design policies to regulate them. That is, access routes may simply be allowed, or on the contrary, be disincentivized or prohibited altogether. Alternatively, they may be not merely allowed, but actively regulated in order to enhance transparency and promote equal access. In advance, we had developed an argument scheme featuring an overview of the moral arguments for and against allowing these three access models. The aim of our study was twofold. Firstly, we aimed to validate and further develop the argument scheme–aimed to aid policymakers and other stakeholders when designing policy options for ethical access to non-reimbursed treatments–through discussion with a diverse group of stakeholders. And secondly, we aimed to bring together groups of stakeholders with different perspectives, who normally would not discuss the ethics of access together, to facilitate the exchanging of ideas and perspectives, stimulate stakeholders to weigh ethical arguments against each other, and search for common ground. Interaction between stakeholders with varying perspectives is important when discussing policy, as it can bring new arguments to the fore, help deepen a discussion, and make sure all relevant impacts are weighed (OECD, 2021). Ultimately, multi-stakeholder discussions can thus help find common ground and advance the societal discussion on fair access to non-reimbursed medical treatments.

Although this study was performed within the Dutch healthcare system, its results are also relevant for other countries with publicly funded healthcare systems. As governments are grappling with problems concerning limited healthcare budgets and increasingly expensive treatments that could potentially crowd out other types of healthcare, insight into stakeholders’ perspectives regarding the ethics of access to non-reimbursed treatments is highly relevant for all countries with publicly funded healthcare systems.

## 2 Materials and methods

### 2.1 Methodological approach

This study was part of the last phase of a broader empirical bioethics research project regarding the ethics of access to non-reimbursed treatments. In empirical bioethics research projects, roughly 3 phases are distinguished: the phase of *mapping* of the field (for instance with a literature study), the phase of *framing* of the research problem or area (further exploring a specific problem or area, for example, by conducting qualitative interviews) and the phase of *shaping* of the terrain (for instance, by developing normative recommendations for new policies) (Huxtable and Ives, 2019). This study was part of the third phase of our research project, and aims to integrate the empirical work with the normative. Therefore, it does not remain only descriptive of individual stakeholder perspectives, but in bringing varying stakeholders together to exchange different moral perspectives and weigh ethical arguments, it seeks common ground and tries to develop recommendations. There are roughly two kinds of overarching approaches in integrating the empirical and normative work within empirical bioethics: the consultative approach and the dialogical approach (Davies et al., 2015). In the consultative approach, the normative analysis takes place after stakeholders are consulted. The input from stakeholders is collected and analyzed afterwards by the researcher, and normative conclusions are developed after the interaction has taken place–often after consulting ethical theories. In the dialogical approach, normative claims are developed during the interaction with stakeholders, often seeking a shared understanding or consensus (Widdershoven et al., 2009; Davies et al., 2015). In previous studies, we have used the consultative approach, and conducted qualitative interview studies to understand stakeholders’ perspectives (framings) on the ethics of access (Bomhof et al., 2022). As diverse groups of stakeholders are affected by this dilemma, and policies should ideally be supported by these groups of stakeholders, for this study, we have chosen a dialogical approach aimed at shaping the terrain. As methodologies used for integrated empirical bioethics are diverse and often remain inexplicit, researchers within empirical bioethics have been called upon to reflect upon the normative justification and methodological approach used (Davies et al., 2015). With these dialogues, we aim to contribute to the tradition of the dialogical approach, by developing a format in which stakeholders *with diverse backgrounds* could exchange perspectives and weigh moral arguments together, potentially leading to normative common ground or recommendations.

### 2.2 Design of dialogues

Three in-person multi-stakeholder dialogues were held in two meeting centres in Utrecht, a central location in the Netherlands, in February and March 2023. Each stakeholder dialogue included seven to eight purposively selected participants. The meeting rooms had a hollow-square set-up to facilitate interaction between participants. At the start of the dialogues, agreements were made regarding confidentiality and respectful dialogue to create a safe environment. All dialogues were led by the same moderator (MS). Other members of the research team (EB, CB, JS, SS) were also present to take notes, to ask questions for clarification or follow-up, or to answer factual questions from participants. During the dialogues, key considerations were noted on a flip-over (by CB). Each dialogue lasted approximately 4 h in total. Dialogues were audio-taped.

### 2.3 Participant selection

Selection of the participants was done via purposive sampling. In previous (interview) studies and field work (Bomhof et al., 2022; Bomhof and Bunnik, 2023), relevant groups of stakeholders had already been identified. These stakeholders were: hospital managers, health insurers, policymakers, physicians, patients, citizens and relevant professional and representatives of pharmaceutical companies, patient and physician organisations. Participants were approached by email or by telephone. Of each group of stakeholders, one representative was invited for each discussion. For the selection of the citizens, we contacted a market research bureau through which we could approach individuals who had previously attended a citizen panel regarding allocation choices in healthcare (Burgerforum, 2018). This way, we were able to ensure that our citizen-participants had basic knowledge of the Dutch healthcare system and some familiarity with questions regarding the allocation of (scarce) healthcare resources.

### 2.4 Dialogue format

For the design of the format for the stakeholder dialogues, we have drawn inspiration from the nominal group technique (McMillan et al., 2016) and literature on the dialogical approach (Widdershoven et al., 2009; Davies et al., 2015). Our goal was to develop a format in which stakeholders could exchange perspectives and weigh arguments together.

To help prepare for the discussion, all participants received an ‘argument sheet’ which we drafted in advance (see Appendix A). This argument sheet contained an overview of the moral arguments in favor of and against three policy options. One week before the dialogue, participants were asked to share their preliminary perspectives regarding the three policy options in a short online survey (see Appendix B). At the start of the dialogue, one of the research team members (EB) gave a presentation on the CL and the policy options, to make sure that each participant had sufficient background knowledge.

At the start of each dialogue, participants were asked to indicate their normative viewpoints regarding the three policy options, indicating for each policy option with a sticker on a poster (see Appendix C) whether they were “very much against” “against” “neutral” “in favor” or “very much in favor” of this policy option. Subsequently, three discussion rounds were held of approximately 1 h each. In each round, one policy option regarding access to treatments in the CL was discussed. Each discussion round was divided into three phases:

1) All participants briefly shared their perspectives regarding the policy option. Other participants could ask questions for clarification, but could not yet respond substantively to each other’s arguments
2) A general discussion took place in which participants were asked to exchange views and invited to elaborate on their positions and question the perspectives of others.
3) In a final round, participants were asked to evaluate and weigh the arguments, to gauge whether or not participants had shared key considerations about the policy option.

After the three discussion rounds, participants were asked once more to indicate their normative viewpoints regarding the three policy options by putting a sticker on the poster. This way, we could determine whether stakeholders had shifted. Every dialogue was closed off with a round of reflection in which the participants gave feedback on the proceedings and shared whether they had heard any arguments that had led them to change their opinion.

### 2.5 Data analysis

The three audio-taped dialogues were transcribed in Word and coded using Word and NVIVO. All transcripts were independently coded using an inductive approach (by SS and CB/JS). During the coding process, weekly meetings were held with the research team to discuss the coding and straighten out discrepancies, and develop the codebook. A thematic analysis (Burgerforum, 2018) was conducted. A inductive approach was used to identify relevant themes. Both recurring overarching themes and themes per model were identified.

### 2.6 Ethical approval and informed consent

A waiver for this study was granted by the research ethics review committee of Erasmus MC, University Medical Centre Rotterdam (MEC-2020–0828), as the study does not fall within the scope of the WMO (the Dutch Medical Research Involving Human Subject Act).

## 3 Results

### 3.1 Sample

Of the approached stakeholders, representatives of one professional association and one pharmaceutical company did not wish to participate. Two approached patient representatives were not available at the time the dialogues were to be held, and were replaced by others. On the day of the first dialogue, a health insurer and a representative of a sector organisation had to cancel because of illness or personal circumstances. On the day of the second dialogue, the same two stakeholders had to cancel again. An overview of the participants attending the dialogues can be found in Table 1.

**TABLE 1:** Overview of the participants attending the multi-stakeholder dialogues.

Dialogue 1	Dialogue 2	Dialogue 3
1 doctor	1 doctor	1 doctor
1 citizen	1 citizen	1 citizen
1 insurer	1 insurer	1 insurer
1 patient representative	1 patient representative	1 patient representative
1 policymaker	1 policymaker	1 policymaker
1 representative of pharmaceutical companies	1 representative of pharmaceutical companies	1 representative of pharmaceutical companies
1 representative of medical professional organization	1 hospital manager	1 representative of medical professional organization
		1 hospital manager

### 3.2 Themes

In this section we will first present the main findings per model and then discuss four overarching themes that surfaced during the dialogues; 1) weighing of individual interests *versus* collective interests, 2) shifting attitudes when confronted with other perspectives, 3) withholding access to put pressure on the system and 4) the importance of transparency regarding the CL-procedure. In the Results section, the perspectives of participants are presented to the extent that they are relevant to describe the weighing of ethical arguments. Relevant quotes can be found in Table 2.

**TABLE 2:** Participant quotes.

Quote	
Q1	*‘…. I think…. that the Coverage Lock is meant to assess whether treatments are effective or cost-effective, and they’re almost always effective, but almost never cost-effective. And then I think that we should not pay for those [treatments] from public resources if cost-effectiveness is not yet established. So then the pharmaceutical company should do it [fund these treatments].’* Participant dialogue 1
Q2	*‘I think it all comes down to equality. Everything [every treatment] that someone [some physician] has to search for or negotiate for, it leads to inequality. Physicians work in different shifts and have different motivations, know different things. So I believe it leads to inequality, and that’s not fair. And besides that, I think that it comes down to using public resources, as it costs time, and this time comes from public resources.’* Participant dialogue 1
Q3	*‘Patients really want access, but I think I would go for the principle of equality here, which I believe is very important, that patients have equal opportunities. And again, we do not have that [equality] in the Netherlands, but we should not go and increase it [inequality] either. And if you allow patients to pay themselves, then some patients can do so and others cannot.’* Participant dialogue 1
Q4	*‘People do not choose to have a low life expectancy because of their socioeconomic status. There is so much inequality already. Also regarding assertiveness and how well one knows one’s way around in healthcare–I think everyone knows examples from their own social circle–I think that [inequality] is undesirable.’* Participant dialogue 2
Q5	*‘It is my own money. So I should be allowed to decide whether I want to use it for my health or not. Right? It would be very strange if the government dictates that you cannot use your own money for your own health.’* Participant dialogue 3
Q6	*‘Who are we to decide for someone else [that he may or may not save himself]…. we are talking about effective treatments (…), who are we then to all decide that I will swing? [that I will die]?*’ Participant dialogue 3
Q7	*‘It is very complicated. Actually the same as just discussed: People with money can afford it [paying for treatments]. I think, it [paying for treatments] should be allowed, but then what do we do with people who cannot afford to pay [for treatments].’* Participant dialogue 2
Q8	*‘Yes, well, what you just mentioned about that neuroblastoma: if it concerns young children and it in fact looks like the treatment is effective [they should have access to that treatment].’* Participant dialogue 2
Q9	‘*It is the same as [another participant] said: Every patient has the right to use all resources to save his own life… [Another participant answers] Well, maybe it is about the individual* versus *the collective.’* Participant dialogue 3
Q10	*‘In principle I would say no [to making exceptions]: you cannot do that, if, on the one hand, you uphold a system based on solidarity, and on the other hand, you make these [exceptions] possible, that just leads to inequality, and is completely inhuman. But indeed, maybe that does not matter if it concerns young children and [the treatment] is potentially life-saving or something. But on the other hand, I think, those are exceptions and if those become the rule, then what kind of system are you left with*’ Participant dialogue 2
Q11	*‘In general, it seems to me that it would undermine the solidarity-based system. [Mentions a case of a young girl with neuroblastoma]. So I struggle with that, and I do not know why in that case, I do think it is appropriate [to provide access]. Maybe because there is a whole life ahead of them, that is the strongest consideration.’* Participant dialogue 2
Q12	*‘It could also be used as a sort of canary in a coal mine. If we would do that [allow out-of-pocket payments], then we really have not organized the system in a right way anymore.’* Participant dialogue 3
Q13	‘*I do believe that there should be a certain degree of transparency. So, from day one in which, in the Netherlands, the first patient gets treated [based on payment by a third party, including the hospital], this should be made very clear. And it should be transparent, including the conditions [for getting access to the treatment].’* Participant dialogue 1

#### 3.2.1 Model 1: access based on third-party payment (e.g., by pharmaceutical companies, health insurers or hospitals)

In relation to model 1, the following four themes emerged during the dialogues: 1) reasons to allow access to treatments in the CL, 2) differences between parties considered for third-party payment, 3) equality and other reasons not to provide access, 4) the role of physicians in pursuing access.

##### 3.2.1.1 Reasons to allow access to treatments in the CL

Participants often felt that the provision of access to treatments in the CL–i.e., by third party-payment–was important, thereby relying on beneficence as an important ethical principle. Participants cited potential health benefits for patients as one of the reasons for wanting to provide access to treatments in the CL. Participants mentioned several situations in which they deemed access to treatments in the CL to be extra important: when patients are young, treatments are highly effective, or no alternative therapies are available. Some participants stated that physicians should be able to prescribe all relevant treatments, including treatments that were placed in the CL. One participant believed that all EMA-approved treatments should be available for patients as a matter of principle. However, participants also frequently mentioned concerns regarding the often-marginal benefits of newly approved treatments, and believed these should weigh in the decision whether to seek alternative access routes for treatments in the CL.

##### 3.2.1.2 Differences between parties considered for third-party payment

During the dialogues, three potential parties for third-party payment were considered: hospitals, insurance companies and pharmaceutical companies. Most participants believed that pharmaceutical companies were a better suited third-party payer than hospitals or insurance companies, as they believed it would be unjust to use *collective* funds to pay for treatments for which (cost-)effectiveness was not yet clear (Q1). Allowing hospitals or insurance companies to pay for these treatments could undermine the role of the CL in guarding against excessive healthcare expenditures. It was felt that the CL helps to prevent expensive treatments from crowding out other forms of healthcare, as well as from usurping public spending outside the healthcare domain, for instance, in education. A second reason against allowing hospitals or insurance companies to pay, was that this use of collective funds (i.e., from hospital or insurance budgets) could weaken the government’s negotiation position during price negotiations with pharmaceutical companies, because there would be less of an incentive for the latter to lower the price. Only in the third dialogue, some participants considered it appropriate if insurance agencies were to pay for treatments in the CL. Reasons given were that insurance companies would also pay for treatments once they come out of the CL, and that it would provide an opportunity for data collection on the effectiveness of these treatments in real-world settings. However, most participants were in favour of letting pharmaceutical companies pay, as pharmaceutical companies would not be using collective funds. Furthermore, some participants mentioned that letting pharmaceutical companies organize managed access programs for all eligible patients was the only way of ensuring equal access to treatments in the CL. However, it was noted that in practice, access to treatments would then solely depend on the willingness and ability of pharmaceutical companies to pay, which might result in limited or variable availability of CL treatments. To safeguard equal access, it was deemed important that payment by pharmaceutical companies would not be organized for individual patients, but–solely–through managed access programs open to all eligible patients. Furthermore, participants pointed out other (adverse) effects of allowing pharmaceutical companies to pay for treatments; firstly, pharmaceutical companies might use these programs to expand their post-CL sales opportunities. Secondly, pharmaceutical companies might account for money spent on CL-treatments during price negotiations. However, this might imply that ultimately, society ends up paying more for these treatments. Thirdly, the negotiation position of pharmaceutical companies would be undermined if they provided access for all patients while treatment are in the lock, at least in the absence of set procedures that limit the duration of the negotiation.

##### 3.2.1.3 Equality and other reasons not to provide access

Participants also voiced concerns regarding third-party payment in general, sometimes emphasizing the importance of equal access for patients to treatments over that of individual benefits. Participants feared that third-party payment might potentially lead to arbitrariness in hospital-based decision-making about patients access to CL-treatments. They believed that patient access should not vary between hospitals or physicians, and that all eligible patients should be able to get access to relevant treatments equally. Some participants also mentioned that patient access to reimbursed forms of healthcare is currently unequal, at times, due to practice variation, and therefore wondered whether equal access to non-reimbursed treatments in this model would be a utopia. Participants also emphasized the role of the CL in safeguarding the sustainability of the healthcare system, raising concerns that all sorts of third-party payment might potentially undermine society’s efforts to ensure cost-effectiveness in the allocation of healthcare resources.

##### 3.2.1.4 The role of physicians in pursuing access

During the first two dialogues, participants also deliberated on the role physicians should play in arranging access to treatments based on third-party payment. Many participants believed that physicians should not try to arrange access as this could lead to practice variation amongst physicians and therefore enhance inequalities in patient access (Q2). For instance, some physicians might be more willing to spend time and energy pursuing treatment access or have a better network or negotiating capacities than others, giving them more opportunities to arrange access for their patients. Secondly, participants believed that arranging access to non-reimbursed treatments should not be a part of the range of duties of physicians and physicians should focus on their regular care duties. Thirdly, as arranging access costs time, participants said, it could potentially lead to physicians having less time available for other patients, thus crowding out healthcare for others (Q2). However, some participants believed that physicians should try to arrange access, mainly because of the potential health benefits for patients. One participant felt that physicians *should* pursue access if a treatment were highly effective and would lead to significant health benefits for patients. Some participants called for transparency and clear guidelines for physicians regarding whether and when to pursue access to CL-treatments. One participant mentioned that professional associations, for example, of hematologists, should try to arrange access instead of individual physicians.

#### 3.2.2 Model 2: Access based on out-of-pocket payments by patients

During the discussion of model 2, participants more explicitly mentioned ethical values which they believed were at stake, namely, justice, solidarity, non-maleficence and liberty.

##### 3.2.2.1 Justice

Many participants addressed concerns regarding out-of-pocket payments increasing inequality amongst patients, as some patients would be able to pay for treatments while others would not, and therefore, allowing patients to pay was seen as unjust (Q3). Participants also mentioned that this inequality would not be ‘at random’ but would enhance pre-existing structural inequalities between citizens based on socio-economic status. Participants deemed equal access in healthcare to be very important. For some participants, equal access for all patients outweighed potential health benefits for individual patients. Some participants pointed out that inequality would also be enhanced in the case of crowdfunding, as some patients will have a better social network and social and financial resources to start successful crowdfunding campaigns than others. This would also exacerbate an existing divide on the basis of differences in socio-economic status (Q4). Conversely, two participants mentioned that for them, the fact that inequality already exists in the Netherlands was a reason not to consider inequality to be an important argument, especially as access to CL-treatments was seen as rare. Others responded that these existing inequalities are problematic as well, and are no justification to allow further inequalities.

Another form of injustice mentioned by participants was that, as said, out-of-pocket payments could potentially lead to the crowding out of other health services within the publicly funded healthcare system. If patients paid for treatments out of pocket, physicians would still spend time administering these treatments and patients would need follow-up care in the case of adverse events–potentially occupying hospital beds or staff, leaving less capacity for others. Some participants mentioned that patients could be allowed to pay for treatments out of pocket, but they should then also pay for any ancillary costs and additional medical care to prevent this scenario from happening.

##### 3.2.2.2 Solidarity

Solidarity was another value frequently mentioned by participants regarding out-of-pocket payments. Participants believed that allowing out-of-pocket payments would be undermining the solidarity-based healthcare system, which would be undesirable. In addition, in the first dialogue, participants wondered whether out-of-pocket payments could lead to a shift in *perceptions* of the Dutch healthcare system: people might come to think of healthcare as purchasable and on the long-term this would lead to a decrease in experienced solidarity in healthcare in the Netherlands.

##### 3.2.2.3 Non-maleficence

During the first dialogue, participants were concerned that not allowing out-of-pocket payments could potentially lead to patients travelling abroad to obtain these treatments. They considered this potentially harmful, as standards of care in other countries might be lower than those in the Netherlands. Nevertheless, in general, participants believed that arranging access in the Netherlands fairly, was more important than considering the harms for patients travelling abroad. However, participants also feared that *allowing* out-of-pocket payments could also be potentially harmful; namely, leading to financial harms if patients were to spend large amounts of money on expensive treatments.

##### 3.2.2.4. Liberty

In all dialogues, participants mentioned liberty as an important value when considering out-of-pocket payments. Some participants mentioned that although they felt that equality was important, it was not deemed possible–or, by some participants, not deemed desirable–to forbid patients to pay for treatments out of pocket, placing more emphasis on the value of liberty (Q5). Participants mentioned that people should maintain their freedom to spend their money as they seem fit. Some participants believed that forbidding patients to pay for treatments, especially if these treatments could lead to significant health gains, would go ‘too far’. During the third dialogue, the argument of liberty explicitly came to the fore, when a patient-representative spoke about the feeling of fear he experienced when an effective treatment for his disease was placed in the CL. This participant had wanted the freedom to arrange access to this treatment himself, if necessary (Q6). During the dialogue, other participants expressed empathy for this reasoning. However, participants were conflicted when having to weigh liberty against equality, since equal access was also deemed to be essential by many participants (Q7).

#### 3.2.3 Model 3: No access to non-reimbursed treatments

During the third discussion round, it was notable in each of the three dialogues, that participants seemed to naturally–without extensive discussion - come to conclusions as to whether they believed access to non-reimbursed treatments should or should not be possible.

##### 3.2.3.1 Access should be possible

A majority of the participants thought that it should be possible for physicians and patients to pursue access in one way or another (for instance through third-party payment), if physicians believed that a treatment was truly in a patient’s best interest. In such cases, participants felt it was important that physicians should retain the possibility to pursue access for individual patients–to look for ‘shortcuts’, such as submitting individual requests to pharmaceutical companies or insurance agencies. Others believed that in such cases, access should be made possible for *all* patients–for instance through a managed access program–to ensure equal access. Participants mentioned multiple exceptional circumstances in which they believed patients should have access to treatments in the CL. Criteria which were mentioned for making such exceptions were: if patients are young, if patients are severely ill, if no alternative therapy is available and if the treatment seems highly effective with large potential health gain for patients (Q8). Some participants also stressed that the CL is a means for guarding against insufficiently cost-effective use of collective funds, and not a goal in itself. Therefore, they believed that patients should not experience harm resulting from not being able to access treatments placed in the CL.

##### 3.2.3.2 Access should not be possible

Some participants believed that it would be the fairest option if no-one ever had access to treatments while they were in the CL. These participants stressed that this was the most equal option, again underlining the importance of equality regarding access to healthcare. Some participants also mentioned that this option would provide the most transparency and clarity for all relevant stakeholders, including pharmaceutical companies, patients, and healthcare professionals.

#### 3.2.4 Overarching themes

##### 3.2.4.1 Weighing individual interests against public interests

In all dialogues, tensions between individual interests and collective interests were a recurrent theme. In all dialogues, considerations regarding individual and collective interests came hand-in-hand with debates on liberty and solidarity. On one hand, participants mentioned arguments that put the individual in the centre, for instance when the argument of freedom to spend one’s own money as one sees fit was recurrently brought to the fore. The interests of the individual were also highlighted when participants elaborated on potential health gains for patients. On the other hand, participants frequently emphasized collective interests, as they stated the importance of solidarity in our healthcare system, and the collective duty to keep the healthcare system sustainable for future generations of patients. During the dialogues, participants often felt conflicted when weighing individual interests against collective interests (Q9).

##### 3.2.4.2 Shifting attitudes

Sometimes, when, during the dialogues, participants brought up a (fictitious) concrete example of a cancer patient who would benefit from access to a treatment in the CL, other participants changed their expressed attitudes towards (dis)allowing out-of-pocket payments for treatments in the CL. Participants were inclined to “make exceptions” for these particular patients. This for instance happened when a case was brought to the fore of a young patient. One participant mentioned that inequality might matter less if the lives of young children could be saved (Q10). Some participants considered this–sometimes internal–shift puzzling and intriguing. One participant was puzzled that he considered it ‘okay’ to allow out-of-pocket payment for a CL treatment for a four-year-old girl with a neuroblastoma, while he had previously stated that he was against out-of-pocket payments (Q11). In another dialogue, participants noted that their weighing of the arguments would change considerably in the consulting room when face-to-face with a patient, especially when the patient-doctor relationship was a longstanding one. However, even without a longstanding relationship, it was considered very difficult for a physician *not* to help a patient arrange access to a treatment.

##### 3.2.4.3 Withholding access to put pressure on the system

In all three dialogues, participants talked about deploying the three models strategically to put pressure on stakeholders involved in the CL-procedure to reduce the time treatments spend in the CL. For example, one participant mentioned that he was in favour of *allowing* out-of-pocket payments because this would be considered politically unacceptable in our society, and the resulting upheaval might lead to acceleration of the CL-procedure. This view was echoed in another discussion, with a participant remarking that out-of-pocket payments could be used as a kind of signal, as a ‘canary in the coal mines’, that the system was failing (Q12).

Pressuring the system to accelerate the CL-procedure was also mentioned as a reason to consider the model in which *no-one* would obtain access to treatments placed in the CL. Participants believed that withholding access would generate societal pressure on pharmaceutical companies and parties involved in the CL-procedure to accelerate the procedure. However, in the third deliberative discussion, one participant believed that this pressure would create much societal turmoil, which would not necessarily help move the discussion regarding the CL-procedure forward. However, others countered this statement and believed that uproar is inevitable in allocation decision-making in healthcare, pointing out that a negative reimbursement decision for a treatment would also cause uproar.

##### 3.2.4.4 The importance of transparency regarding the CL procedure

During the dialogues, many participants stressed the importance of transparency regarding the CL-procedure. This included transparency regarding the results of price negotiations, the time which treatments spend in the lock, and possibilities for patients to access these treatments while they are in the lock. During the discussion of the first model, this last point was emphasized. Participants mentioned that it should be clear and transparent for patients and physicians how and in which hospitals patients can get access to a CL-treatment–for instance, through a managed access program–to ensure equal access to these treatments for patients (Q13). Participants also stated that it should be transparent for whom–for instance which categories of patients–access to treatments in the CL was possible, and how long the CL-procedure would take. This would help prevent societal unrest. Furthermore, participants criticized the current CL-procedure for being non-transparent about price-negotiations and treatment-prices that are eventually agreed upon. Many participants stressed the importance of a clear CL-procedure for all stakeholders–especially patients–to know where they stand. Some participants therefore were in favour of the third model, as it would provide clarity if no patients had access to treatments during the CL-procedure, while ‘making exceptions’ could create uncertainty.

## 4 Discussion

This multi-stakeholder dialogue study regarding the CL-procedure in the Netherlands showed that stakeholders have varying perspectives on access to non-reimbursed treatments. Generally, participants were in favour of allowing access–under specific circumstances–to CL-treatments so as to not withhold potential health gains from patients in need. When discussing third-party payment, participants favoured payment by pharmaceutical companies over payment by health insurers or hospitals, as they considered it unjust to usurp collective funds while cost-effectiveness assessment was still pending. Largely, participants weighed the moral values of solidarity and equal access over the values of liberty and beneficence, and were therefore not in favour of out-of-pocket payments. The publicly funded healthcare system in the Netherlands, with an obligatory health insurance for all citizens and equal access to a basic healthcare package, is strongly based on the values of solidarity and equal access (Zorginstituut Nederland, 2022). During the dialogues, stakeholders emphasized both the importance of these values and the valuable role of policy measures such as the CL in safeguarding the sustainability of the healthcare system.

### 4.1 Individual *versus* collective interests

During the dialogues, four over-arching themes emerged which require ethical reflection. Firstly, it may be difficult to weigh the interests of individual patients against those of the collective in the context of access to non-reimbursed treatments. Treating physicians may need to help eligible patients gain access to treatments in the CL because of potential health benefits. This would be in line with the principle of beneficence: a physician’s obligation to act to the benefit of patient (Beauchamp and Childress, 1979). In addition, one would like to allow individual patients the freedom to spend their money on medical treatments that might otherwise not be accessible, if they can and wish to do so. However, it is unclear how beneficence and liberty should be weighed against the importance of sustaining an equitable and solidary healthcare system in a country like the Netherlands.

These tensions between individual and collective interests are reflected in a recent analysis of the concept of solidarity. Solidarity may refer to various sets of norms: assisting patients in need; upholding the solidarity-based healthcare system; willingness to contribute; or promoting equality (van Till et al., 2023). In the context of (dis) allowing out-of-pocket payment for CL treatments, for instance, helping patients (crowd) fund medical treatments, can be seen as an act of solidarity on the individual or inter-individual level, but it can also be seen as undermining solidarity on a societal level, by jeopardizing the sustainability of the healthcare system or failing to promote equality. In addition, if one were a–more affluent–patient oneself, and chose to pay out of pocket for CL treatments, leaving other–less affluent–patients behind, one would be considered a failure to show solidarity towards these others patients. The results of our dialogues suggest that while stakeholders may perceive collective interests to be important, they may sometimes let individual interests outweigh collective interests, and that–at least in specific circumstances–stakeholders support patient access to treatments in the CL.

### 4.2 Shifting attitudes and the identifiable victim effect

Secondly, it was notable that sometimes a shift in attitudes–or expressed opinions–occurred when stakeholders discussed (fictitious) patient-cases. When confronted with detailed information about (fictitious) individual patients, participants would nuance their expressed opinions on not allowing access to treatments in the CL, and become more inclined to ‘make an exception’ for these particular patients. This could be explained by the so-called identifiable victim effect and the rule of rescue. According to the identifiable victim effect, people are more likely to help an identifiable victim than a statistical victim (Jenni and Loewenstein, 1997). Relatedly, according to the rule of rescue, people have a strong moral inclination to rescue the lives of *identifiable* persons in immediate danger (Jonsen, 1986). This could explain why, during the dialogues, stakeholders could discuss access models on the level of the population or healthcare system in general terms, referring to probabilities and numbers, but when concrete (fictitious) patient-cases were brought up, they changed their expressed opinions. This manifested itself clearly in the third dialogue, when a patient representative spoke about his own experiences, stating that no-one at the table truly understood what it meant to be ill and not to have access to a potentially life-saving treatment in the CL. From then onwards, participants adjusted their–at least expressed–opinions, expressing their sympathy and reasoning more in favour of access. Many participants seemed sensitive to the emotional appeal made by a patient case.

In the literature, there are ongoing debates on the merits and pitfalls of the identifiable victim effect and the rule of rescue (Daniels, 2012; Victoria, 2022). It is important to be aware of these *effects* in discussions on policy options, as they can potentially obstruct the consideration of collective interests and the equal accounting of unidentifiable victims in decision-making. As the patient perspective ought to inform decision-making, it is important that policymakers are aware of these effects, to minimize the chance of collective interests–including upholding a sustainable public healthcare system–being underrepresented in the development of policy.

### 4.3 Call for strategic action and transparency

The third and fourth overarching themes were closely associated. Both the call to use the models strategically to accelerate the CL-procedure and the call for more transparency regarding the CL-procedure stemmed from dissatisfaction with some aspects of the current CL-procedure. Criticism of the lack of transparency and the duration of the CL-procedure has also been found by Scheijmans et al., in their study on the experiences of stakeholders during the CL-procedure of Nusinersen (Scheijmans et al., 2022). The lack of transparency is problematic from the perspective of procedural justice. Like most priority setting agencies, the Dutch Healthcare Institute explicitly aims for just procedures for priority setting, adopting the Accountability for Reasonableness framework by Daniels and Sabin (Daniels, 2008; Zorginstituut Nederland, 2017) The first condition of the framework, the ‘publicity condition’, states: “decisions regarding both direct and indirect limits to care and their rationales must be publicly accessible” (29, p.45). A major obstacle in this regard is the fact that the results of price negotiations remain undisclosed, which makes it impossible for parties other than the government and pharmaceutical companies to evaluate the reasonableness of decisions (not) to include a new treatment in the basic benefits package. Closely related is the lack of public insight into the reasons and causes of a long duration of the negotiations, for this makes it impossible to know which party (i.e., the government or the pharmaceutical company) should be held accountable for keeping patients waiting. Thus, the publicity condition, at present, remains unfulfilled.

### 4.4 Methodological reflection

With this study, we present a format for engagement between a diverse group of stakeholders aimed at exchanging different moral viewpoints and seeking normative common ground. Drawing inspiration from the nominal group technique (McMillan et al., 2016) and dialogical approach (Widdershoven et al., 2009; Davies et al., 2015), during the dialogues, we put emphasis on stakeholders to weigh moral arguments together. To do so, prior to the dialogues, we had sent participants an argument sheet detailing relevant moral arguments to help participants form their individual viewpoints in advance. Based on the nominal group technique, in which participants vote for various policy options, we asked participants to express a normative viewpoint regarding each policy option by placing a sticker on a Likert scale on a poster. By doing so, we were able to gain an impression whether consensus was reached, or whether shifts in individual normative viewpoints had taken place. During the dialogues, participants deliberated the different policy options and weighed the arguments pro and con. However, it proved difficult to ask stakeholders to develop *common* normative ground. During the dialogues, we have asked whether–and on which views–participants agreed, but we did not continue to steer towards reaching a consensus if stakeholders had opposing views. This study can be characterized as a normative policy orientated bioethics (NPOB) project (Ives and Draper, 2009), as it tried to integrate empirical findings from our previous research with normative recommendations of stakeholders by using a dialogical approach. As mentioned above, the aim of this study was to see whether multi-stakeholder dialogues could be used to exchange viewpoints and seek for common normative ground. In our study it proved difficult to arrive at definite normative conclusions or consensus. However, whether consensus should be the ultimate goal of such dialogues is questionable, as in a pluralistic democracy, reasonable pluralism–a plurality of reasonable, though irreconcilable moral views–seems a given (Rawls, 1993). So while during the dialogues there was no consensus on the moral dilemma of whether to allow access to non-reimbursed treatments, which was neither expected nor perhaps necessary, stakeholders did arrive at some common normative ground regarding procedural aspects and recognition of certain values. For instance, the importance of taking into account both collective and individual interests, and of the transparency of access routes to non-reimbursed treatments. In the field of empirical bioethics, there is some debate on whether dialogue can be used to derive normative conclusions. Some believe that consensus can have some moral authority, for instance in the context of clinical decision-making (Walker and Lovat, 2022). While we believe that consensus alone does not constitute sound ethical conclusions, this study shows that a dialogical approach can be useful to deepen moral viewpoints and gain a better understanding of moral dilemmas based on the perspectives of stakeholders (Widdershoven et al., 2009). Furthermore, it can help stakeholders in enriching their moral perspectives and understanding the perspectives of others which might help them in decision-making. With this, we might reach the limits of what normative ethics can achieve in a situation of reasonable pluralism and opposing moral views. For future research, it would be interesting to investigate the role of political philosophy, such as deliberative democracy (Rawls, 1993), to integrate the empirical and normative and support political decision making in the face of deep seated reasonable moral pluralism. Furthermore, it would be interesting to asses in which extent the outcomes of such dialogues influence stakeholders’ decision-making in practice.

### 4.5 Strengths and limitations

This study had several strengths and limitations. For this study, we were able to bring together many different stakeholders, including hospital managers, health insurers, policymakers, physicians, patients, citizens, and representatives of pharmaceutical companies, relevant professional, and patient organizations in the field. This resulted in the presence of a broad range of views, helping participants to discover different perspectives and learn from other stakeholders. Furthermore, the dialogues were held in a safe and confidential environment, which had a positive influence on the exchange of views. In total, three dialogues were conducted, to prevent the potential occurrence of groupthink to influence the results. We observed that there were no substantial differences in themes across the three dialogues. We explicitly wanted to involve–informed–citizens in the dialogues, and have tried to provide an equal minimum of background knowledge by providing information beforehand and starting each dialogue with a presentation. However, during two of the three dialogues, citizens were sometimes less involved in the discussion as other stakeholders, as differences in background knowledge were still present. The duration of the dialogues (4 hours) gave room for an extensive exchange of views, and at the end of the dialogues, no new themes emerged. However, some participants mentioned they would have preferred to discuss the topic further–including alternative policy options.

## 5 Conclusion

These multi-stakeholder dialogues emphasize the importance of stakeholder engagement in policy development regarding the sustainability of the healthcare system. Dialogues between stakeholders with varying perspectives on access to treatments in the CL have proved useful to help validate and deepen the moral arguments for and against three models for access to treatments in the CL. Generally, most stakeholders were in favour of allowing access–at least when treatments are clearly beneficial–to treatments in the CL. When discussing third-party payment, stakeholders favoured payment by pharmaceutical companies over payment by health insurers or hospitals, not wanting to usurp collective funds while cost-effectiveness assessments are still pending. Largely, stakeholders were not in favour of out-of-pocket payments, emphasizing solidarity and equal access as important pillars of the Dutch healthcare system. In order to safeguard equal access as much as possible, various stakeholders stressed the importance of transparency as to in which hospitals CL treatments are available by means of managed access. In addition, the call was made for clear and consistent procedures to ensure such transparency, and to reduce the administrative workload for physicians, also in order to prevent displacement of care resulting from excessive burdens on physicians. Policies for access to non-reimbursed treatments should address stakeholders’ concerns regarding transparency, equal access and solidarity, and potential loss of health benefits for patients. Multi-stakeholder dialogues are an important tool to help inform policy-making on access to newly approved, expensive treatments, in the Netherlands, and in other countries dealing with the growing challenges that these treatments pose to the sustainability of the healthcare system.

## Data Availability

The original contributions presented in the study are included in the article/Supplementary Material, further inquiries can be directed to the corresponding author.
