# Supplementary material for: Ethics of access to newly approved expensive medical treatments: multi-stakeholder dialogues in a publicly funded healthcare system
Source: Front Pharmacol. 2024 Jan 29;14:1265029. doi: 10.3389/fphar.2023.1265029 (PMC10863042; doi:10.3389/fphar.2023.1265029)
Supplement: Supplementary file 1 [file Table1.docx]

### **Appendix A: Overview of arguments for and against the access routes to treatments placed in the Coverage Lock**

|  | **Access routes to treatments placed in the Coverage Lock (CL).** | **Arguments pro** | **Arguments contra** |
| --- | --- | --- | --- |
| 1. | As long as a treatment is placed in the CL, no one gains access | - Equal access when in equal need of receiving medical care - Prevents a visible societal divide - Is consistent with the Dutch egalitarian professional ethos in health care | - Limits the liberty to spend one’s own means - Loss of health (people miss out of treatments that potentially improve their health, prolong their lives, or sometimes even cure them from their serious or life-threatening disease) |
| 2 | A third party is allowed to pay | - Treatments can be life-prolonging or sometimes even curative (this applies to 2a-c) | - Leads nearly always to unequal access when in equal need of receiving medical care, also due to differences in assertiveness, knowledge, and relations (applies to 2a-c) - May lead to displacement or delay of necessary care to other patients, due to the deployment of personnel and resources (applies to 2a-c) |
|  | 2a. Insurer is allowed to pay | - Insurers are commercial parties that are free to choose to fund individual treatment requests^[[1]](#endnote-1)^ | - Insurers fulfill a public role^[[2]](#endnote-2)^ and which care is insured should be the same for everyone |
|  | 2b. Pharmaceutical company is allowed to pay | - Pharmaceutical companies are commercial parties that are free to fund individual treatment requests - Generate real-world data and gain clinical experience with the treatment | - Companies can employ an early access program for creating political pressure to ensure inclusion in the basic benefits package |
|  | 2c. Funding from the hospital budget | - Possibility for customization in cases where effective treatment is not reimbursed - Some hospitals have sufficient means, such that there is no displacement of other care | - Unequal access to publicly funded care, to which citizens have equal entitlements, because some hospitals use their own budget, whereas others do not |
| 3. | Patients are allowed to pay out-of-pocket | - Treatments can be life-prolonging or sometimes even curative - It is better to give some patients a chance for health benefits than to give that chance to none - Liberty to spent one’s own means - Physicians and other healthcare professionals can help their patients, which prevents the moral distress resulting from being disallowed to administer effective treatments | - Unequal access when in equal need of receiving medical care - Undermines the strong egalitarian professional ethos in health care and can lead to moral distress for healthcare professionals - Displacement of care that is included in the basic benefits package (due to scarcity of healthcare professionals and beds) - Harm to the patients themselves (financial toxicity, e.g. because they sell their house) Visible inequality may be painful to patients who cannot pay out-of-pocket |

###

### A further elaboration of some arguments

| **Equal access when in equal need of receiving medical care**  Equality and solidarity are core assumptions and core values of the Dutch healthcare system. The young pay for the old, the rich for the poor, and the healthy fort the ill, such that everyone in the Netherlands has equal access to medically necessary care. | **Unequal access** **due to differences in assertiveness, knowledge, and relations is unfair**  Especially in case of access via leniency arrangements with an insurer or pharmaceutical company, or in case of funding from the hospital budget, it is possible that assertive and higher educated patients who know the system manage to achieve more, involving their physician, than other patients. Permitting these access routes therefore increases inequalities based on these patient differences. |
| --- | --- |
| **The egalitarian ethos in health care and society**  The core values equality and solidarity are firmly supported by many healthcare professionals^[[3]](#endnote-3)^ and Dutch citizens.^[[4]](#endnote-4)^ Visible inequalities in access, e.g. between rich and poor patients, contradict that ethos and therefore can lead to moral distress from healthcare professionals and citizens. A visible divide can do harm to the ideal of a society in which citizens relate to each other as equals. On the other hand, being disallowed to treat a patient that pays out-of-pocket may also lead to moral distress for physicians and some citizens will have trouble with such policy. | **Displacement**  Displacement happens at least at the following three levels. First, the governmental budgets are finite, so when more is spent on health care so as to include all new expensive treatments in the basic benefits package, the Netherlands can spend less on education, national safety, housing, etc. The societal causes are then displaced by health care.  Second, displacement within health care can operate via funding. Continuing to fund expensive treatments will be at the expense of other medical care. Alternatively, expanding the budget for expensive treatment or specialist medical care can be at the expense of, for example, mental healthcare for youth, or nursing home care.  Finally, displacement may operate at the level of available personnel and beds. For example, when prescribing a treatment funded by out-of-pocket payment, means that other patients have to wait (longer) for their publicly funded treatment. |
| **Liberty to spend one’s own financial means & loss of health**  Not being permitted to use one’s own financial means to promote one’s health is a serious limitation of the individual liberty of citizens. Therefore, this restriction is in need of justification. Health is sometimes seen as a special good, the distribution of which should not depend on individual financial means. However there are many ways in which financial means can lead to better health, ways which although sometimes undesirable, are not restricted. For example, there is a clear relation between level of education and health.  A general ground for restrictions to individual liberty is the prevention of harm to third parties. In this case, displacement of health care is the most important example of potential harm to third parties that results from permitting out-of-pocket payment. |  |

###

### **Footnotes & cited literature**

Bomhof, C. H. C., Schermer, M., Sleijfer, S., & Bunnik, E. M. (2022). Physicians’ Perspectives on Ethical Issues Regarding Expensive Anti-Cancer Treatments: A Qualitative Study. *AJOB Empirical Bioethics*, *0*(0), 1-12. https://doi.org/10.1080/23294515.2022.2110963

Gronde, T. van der, Groot, C. A. U., & Pieters, T. (2017). Addressing the challenge of high-priced prescription drugs in the era of precision medicine: A systematic review of drug life cycles, therapeutic drug markets and regulatory frameworks. *PLOS ONE*, *12*(8), e0182613. https://doi.org/10.1371/journal.pone.0182613

Maarse, H., & Bartholomée, Y. (2007). A public–private analysis of the new Dutch health insurance system. *The European Journal of Health Economics*, *8*(1), 77-82.

1. In addition to their legal duty to guarantee care that is included in the benefits package of the basic health insurance. [↑](#endnote-ref-1)
2. (Maarse & Bartholomée, 2007) [↑](#endnote-ref-2)
3. (Bomhof et al., 2022) [↑](#endnote-ref-3)
4. https://www.scp.nl/publicaties/monitors/2019/03/29/burgerperspectieven-2019-1 (p47). *(in Dutch)* [↑](#endnote-ref-4)
