# Supplementary material for: Ethics of access to newly approved expensive medical treatments: multi-stakeholder dialogues in a publicly funded healthcare system
Source: Front Pharmacol. 2024 Jan 29;14:1265029. doi: 10.3389/fphar.2023.1265029 (PMC10863042; doi:10.3389/fphar.2023.1265029)
Supplement: Supplementary file 2 [file Table2.DOCX]

**Appendix B - survey**

| What, according to you, are the most important moral considerations regarding option a (Case Lock (CL) treatment available to no one)? |
| --- |
| What, according to you, are the most important moral considerations regarding option b (allowing third parties (pharmaceutical companies, insurers, hospitals) in exceptional cases to pay for CL treatments for individual patients)? |
| Does it matter to you which third party pays in case of option b (allowing third parties (pharmaceutical companies, insurers, hospitals) in exceptional cases to pay for CL treatments for individual patients)? Which third party should be allowed to pay, according to you? More than one answer is possible [pharmaceutical companies, insurers, hospitals, or none of these three]. |
| Please explain your answer |
| What are, according to you, the most important moral considerations regarding option c (allow patients to pay out-of-pocket)? |
| Which policy option do you favour the most? [CL treatment available to no one; allowing third parties (pharmaceutical companies, insurers, hospitals) in exceptional cases to pay for CL treatments for individual patients; allow patients to pay out-of-pocket] |
| Please explain your answer |
| Do you concieve of any other policy options? |
| Which further considerations would you perhaps like to share with us in advance? |
