# Supplementary material for: Ethics of access to newly approved expensive medical treatments: multi-stakeholder dialogues in a publicly funded healthcare system
Source: Front Pharmacol. 2024 Jan 29;14:1265029. doi: 10.3389/fphar.2023.1265029 (PMC10863042; doi:10.3389/fphar.2023.1265029)
Supplement: Supplementary file 3 [file Table3.docx]

**Appendix C - posters**


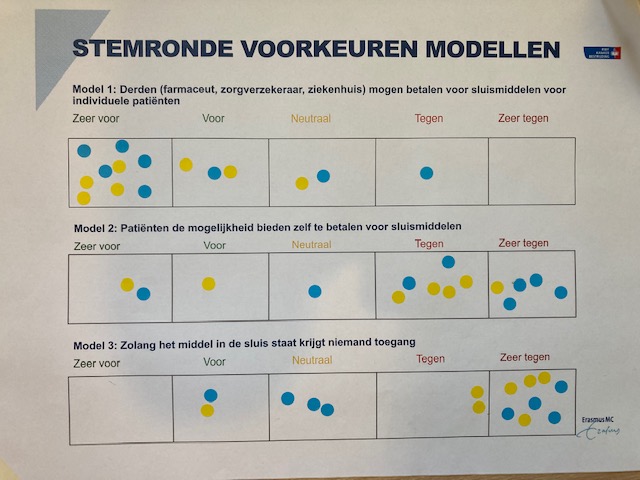


First round: blue, second round: yellow


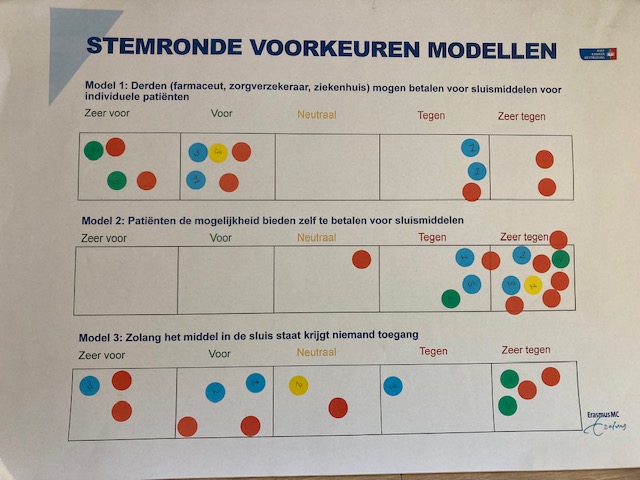


First round: green, blue and yellow, second round: red


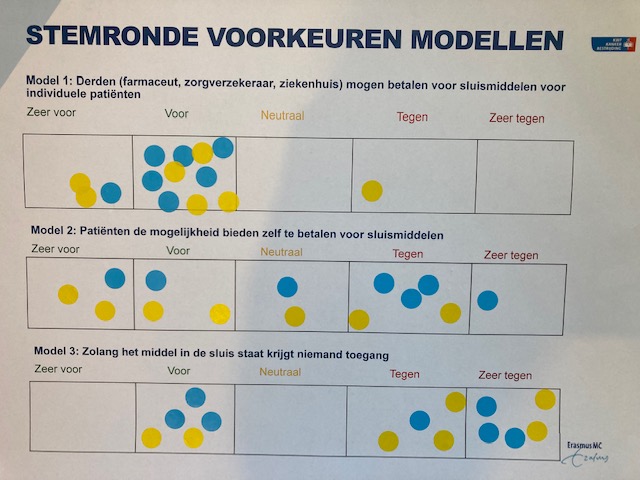


First round: blue, second round: yellow
